# Formation and reshuffling of disulfide bonds in bovine serum albumin demonstrated using tandem mass spectrometry with collision-induced and electron-transfer dissociation

**DOI:** 10.1038/srep12210

**Published:** 2015-07-20

**Authors:** Ine Rombouts, Bert Lagrain, Katharina A. Scherf, Peter Koehler, Jan A. Delcour

**Affiliations:** 1 Laboratory of Food Chemistry and Biochemistry, Leuven Food Science and Nutrition Research Centre (LFoRCe), KU Leuven, Kasteelpark Arenberg 20, box 2463, B-3001 Leuven, Belgium; 2 Deutsche Forschungsanstalt für Lebensmittelchemie, Leibniz Institut, Lise-Meitner-Straβe 34, D-85354 Freising, Germany

## Abstract

Thermolysin hydrolyzates of freshly isolated, extensively stored (6 years, 6 °C, dry) and heated (60 min, 90 °C, in excess water) bovine serum albumin (BSA) samples were analyzed with liquid chromatography (LC) electrospray ionization (ESI) tandem mass spectrometry (MS/MS) using alternating electron-transfer dissociation (ETD) and collision-induced dissociation (CID). The positions of disulfide bonds and free thiol groups in the different samples were compared to those deduced from the crystal structure of native BSA. Results revealed non-enzymatic posttranslational modifications of cysteine during isolation, extensive dry storage, and heating. Heat-induced extractability loss of BSA was linked to the impact of protein unfolding on the involvement of specific cysteine residues in intermolecular and intramolecular thiol-disulfide interchange and thiol oxidation reactions. The here developed approach holds promise for exploring disulfide bond formation and reshuffling in various proteins under conditions relevant for chemical, biochemical, pharmaceutical and food processing.

The formation of disulfide (SS) bonds between correct pairs of cysteine (Cys) residues is essential for the folding, activity and stability of many proteins secreted by living cells^1^,^2^,^3^,^4^. SS bonds play an active role in wound healing^5^, determine protein functionality (foaming gelling, emulsifying) in food systems^6^,^7^,^8^,^9^,^10^,^11^ and can affect protein digestibility and allergenicity^10^,^12^,^13^. While incomplete, the above list demonstrates the importance of SS bonds in a wide range of proteins. In living cells, protein SS isomerase catalyzes reduction, oxidation and isomerization of SS bonds^3^. In the absence of enzymes, SS bond formation through Cys oxidation and SS bond reshuffling through thiol (SH)-SS interchange reactions can also take place, especially at alkaline pH^4^,^14^. SS bond scrambling presents technical issues during recombinant expression, isolation and storage of proteins such as some therapeutic agents, antibodies, receptors, hormones, or enzymes^2^,^4^. That SS bond formation and reshuffling occur during food processing which often includes heating steps, and that they are important for food structure is also undisputed. However, not only the number but also the positions of SS bonds determines protein structure, physicochemical stability and biological properties^4^,^15^,^16^. Technological difficulties associated with current methodologies make that it is generally not known which Cys or cystine [(Cys)_2_] residues dominate protein (in)stability.

Recent proteomic approaches increased the understanding of common posttranslational modifications (PTMs) in eukaryotic cells. These include alkylation, glycosylation, phosphorylation, S-nitrosylation and ubiquitination^17^,^18^,^19^. Oxidative modifications of Cys residues induced by reactive oxygen or nitrogen species or catalyzed by metal ions or enzymes have also been studied^20^,^21^, but the study of non-enzymatic SH oxidation and SH-SS interchange remains challenging^22^. Heat-induced SS bond reshuffling in β-lactoglobulin and κ-casein have been explored by comparing tryptic digests of native and heated proteins (before and after reduction with dithiothreitol) using liquid chromatography (LC)-electrospray ionization (ESI)-tandem mass spectrometry (MS/MS) with collision-induced dissociation (CID)^6^,^7^,^23^. However, CID-MS/MS is not ideal for identifying SS-linked peptides^6^,^24^. While traditional CID produces difficult-to-interpret tandem MS spectra of SS-bound peptides and hence requires reduction of SS bonds prior to MS/MS, electron-transfer dissociation (ETD) favors cleavage of the SS bond^25^,^26^,^27^. The use of alternating ETD and CID increases the amount of information derived from peptide fragmentation^28^ but has so far only been exploited to define SS bonds in native proteins^26^,^29^,^30^. The impact of isolation, storage and heating of proteins on SS bond formation and reshuffling has, to the best of our knowledge, never been studied. We here do so using alternating CID/ETD.

Bovine serum albumin (BSA) is a model protein in many studies. It is structurally well characterized, readily available in its native conformational state, useful in immunodiagnostic procedures^31^, cell culture media^32^ and clinical chemistry^33^, and relevant in foods containing bovine milk or meat. The primary structure of BSA was first reported in 1971^34^ and later revised in 1990^35^. BSA contains 583 amino acids of which one Cys and seventeen (Cys)_2_ residues^36^,^37^. The latter are important from a structural point of view^36^. Isolated freeze-dried BSA is sometimes stored for long times at refrigerator temperature. The impact of isolation and storage on the position of SS bonds is not known. Heating in solution forms SS-linked BSA aggregates^37^ and induces gelation^38^,^39^,^40^. Thermal treatment alters the secondary and tertiary structures, denatures the protein, and thereby impacts the availability of Cys and (Cys)_2_ residues^41^. The crevice in which the free SH group of native BSA is located unfolds upon heating^42^,^43^ but the reactivity of the (Cys)_2_ residues during heating remains to be investigated.

Against this background, the aim of this work was to monitor non-enzymatic PTMs involving Cys and (Cys)_2_ during isolation, extensive dry storage and heating of isolated proteins by analysis of thermolytic digests with MS/MS with alternating ETD/CID. Locations of free SH groups and SS bonds in freshly isolated, extensively stored (6 °C, 6 years, dry) and heated (90 °C, 60 min, excess water) BSA were compared to those in native BSA as deduced from its crystal structure^44^,^45^. This work demonstrates how isolation, storage and heating impact the formation and reshuffling of specific SS bonds in BSA. The approach can easily be applied to other proteins that are relevant in a chemical, biochemical, pharmaceutical or food context.

## Results

### Identification of peptides with native Cys and (Cys)_2_ - residues

Locations of the free SH group and the seventeen SS bonds in native BSA have been deduced from its crystal structure^44^,^45^. Cys or (Cys)_2_ residues present in native BSA are further referred to as native Cys or (Cys)_2_ residues. Here, thermolytic digests were analyzed by LC-ESI-MS/MS with alternating ETD/CID. Thermolysin efficiently cleaves highly SS-cross-linked proteins under acidic conditions, which minimize SH-SS interchange and oxidation reactions^14^. The approach allowed identifying twenty-one peptides (two single-chain, nine double-chain and ten triple-chain peptides) with native Cys or (Cys)_2_ residues in serum albumin isolated from bovine blood plasma, further referred to as freshly isolated BSA (Table 1). Together, they contain the Cys residue and fifteen of the seventeen native (Cys)_2_ residues.

In theory, each BSA molecule contains one free SH group, that of Cys-34. Reduced Cys-34 was detected in the single-chain peptides LQQCP and LQQCPFDEH (Table 1**, peptides 1 and 2)**. A peptide containing Cys-34 SS-bound to free Cys was also found (Table 1**, peptide 3)**. Cys-34 can react with free Cys and glutathione^46^, the concentrations of which in blood plasma approximate 3.5 and 1.7 μmol/L, respectively^47^. No BSA peptide with the Cys-34 linked to glutathione was identified.

Of the seventeen SS bonds reported for native BSA, fifteen were detected, some in various SS-linked double- or triple-chain peptides released by thermolysin. For instance, six different SS-linked double-chain peptides containing the Cys-75, Cys-90, Cys-91 and Cys-101 were found (Table 1**, peptides 6-11)**. Two native SS bonds (Cys-315–360-Cys and Cys-359–368-Cys) were not detected, probably due to incomplete hydrolysis. The location of Cys-315 is unfavorable for thermolytic digestion. Thermolysin preferentially cleaves N-terminal to amino acids with bulky and aromatic residues (Ile, Leu, Val, Ala, Met, Phe) but is hindered by amino acids with acidic side chains (Glu, Asp) in C-terminal position. As a result, the shortest peptide which likely results from thermolytic cleavage of BSA and which contains Cys-315, is AEDKDVCKNYQEAKDA (M_r_ 1825.8). MS spectra were thoroughly but unsuccessfully screened for SS-linked triple-chain peptides containing Cys-315 linked to Cys-360 and Cys-359 linked to Cys-368 (Table 1**, Nd)**. We therefore assume that the M_r_ 1825.8 peptide is too large to be detected as part of a triple-chain peptide.

Unequivocal MS/MS fragmentation spectra were obtained for most peptides, except for the double-chain peptide with a SS cross-link between Cys-53 and Cys-62 (Table 1**, peptide 4)** and the triple-chain peptide containing Cys-460, Cys-476, Cys-475 and Cys-486 (Table 1**, peptide 18)**. The levels of these peptides were too low or they co-eluted with too many other peptides. Their Identification was solely based on their molecular mass. The latter was very accurately determined based on *m/z* values at various charge states.

### Identification of peptides with non-native Cys and (Cys)_2_ residues

Cys or (Cys)_2_ residues not present in crystalline BSA are further referred to as non-native Cys or (Cys)_2_ residues. Twenty-two peptides with such Cys and (Cys)_2_ residues were detected (Tables 2 and 3). Altogether, twenty-one different Cys residues were involved. Most peptides with non-native Cys or (Cys)_2_ residues were detected in all samples analyzed (Table 2). Only four were exclusively found in heated BSA (Table 3).

Cys-75 and Cys-391 are involved in SS bonds in native BSA. Still, they were detected as free SH groups in all samples analyzed (Table 2**, peptides 6 and 15)**. Thus, SS bond reshuffling had occurred. We did not localize other non-native free SH groups but do not exclude their existence in some isolated, stored or heat-treated BSA molecules. Two intermolecular cross-links were found: one between two Cys-34 residues and another between two Cys-513 residues (Table 2**, peptides 1 and 18)**. To the best of our knowledge, this is the first MS/MS approach evidence for BSA intermolecular SS cross-links. Eighteen other peptides were detected with non-native SS bonds. Even though it seems impossible to detect all, our screening suggests that a targeted approach including adequate enzymatic digestion allows detecting specific cross-links.

### Cys and (Cys)_2_ residues in freshly isolated BSA

While its crystal structure shows that every BSA molecule contains one free SH group (Cys-34)^44^,^45^, freshly isolated BSA average wise only contained 0.44 free SH groups. As described above, part of the Cys-34 residues in freshly isolated BSA were involved in SS bonds (Table 1**, peptide 3)**. In addition, several peptides with non-native Cys or (Cys)_2_ residues were detected in freshly isolated BSA which clearly pointed to SS bond formation and reshuffling during isolation. For instance, protein purification induced BSA dimer formation. Proof thereof was the double-chain peptide connecting two Cys-34 residues in freshly isolated BSA (Table 2**, peptide 1)**. Thirdly, SH-SS interchange reactions during protein isolation led to at least eighteen peptides with non-native Cys or (Cys)_2_ residues (Table 2).

The extraction of BSA from blood plasma is based on precipitation of other major blood plasma proteins at an ethanol concentration of up to 40%, pH 4.8 and −5 °C. The resulting BSA was completely soluble in water. Alexander and Hamilton^41^ reported that mild treatments expose nine of the thirty-five Cys residues in BSA without rendering the protein insoluble. However, thirteen Cys residues (Cys-34, Cys-62, Cys-75, Cys-123, Cys-167, Cys-168, Cys-176 or Cys-566, Cys-199, Cys-252 or Cys-288, Cys-264, Cys-391, Cys-460, Cys-513) were detected with a free SH group or as part of an SS bond not present in native BSA. It cannot be excluded that even more Cys residues were involved in SS bond formation or reshuffling as a result of isolation. Thus, mild treatments expose at least 35% of the SS bonds of BSA. These Cys and (Cys)_2_ residues may be more susceptible to SH-SS interchange and SH oxidation reactions than other Cys or (Cys)_2_ residues.

### Cys and (Cys)_2_ residues in extensively stored or heated BSA

**Figure 1:**
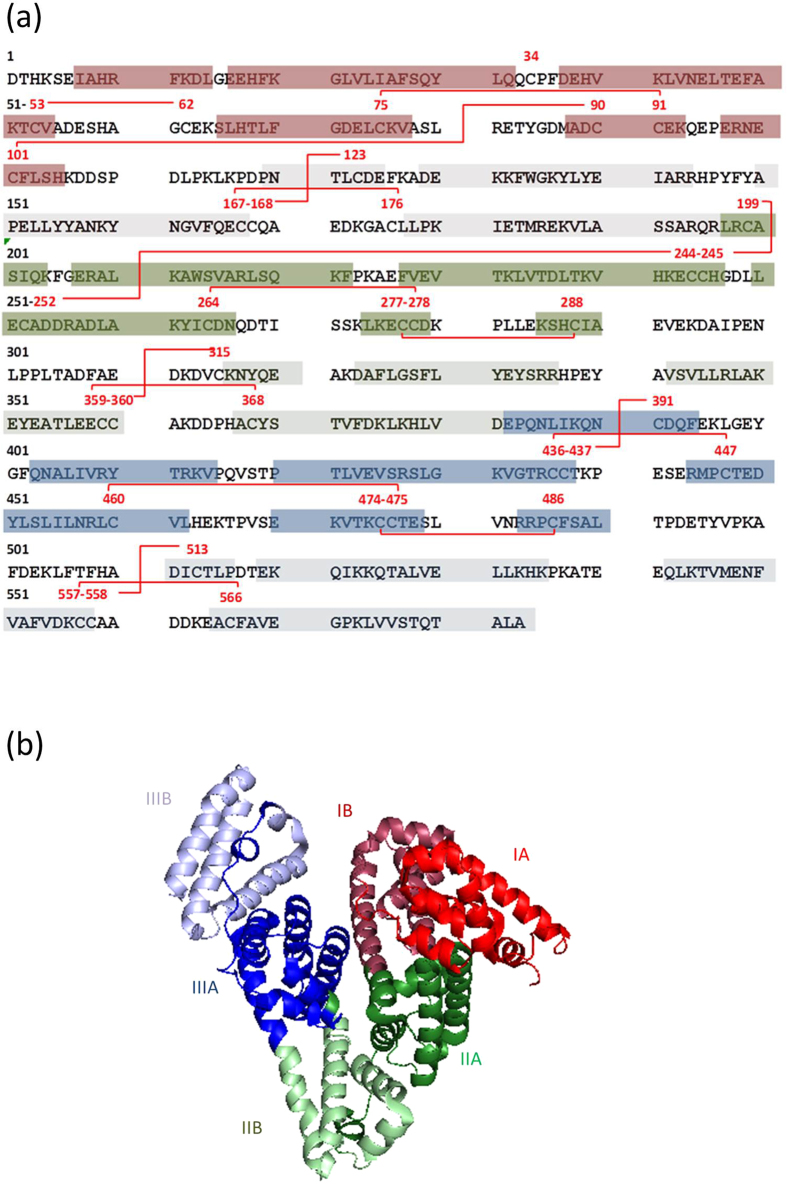
Bovine serum albumin primary (**a**) and secondary (**b**) structures, the latter with indication of its three α-helical domains (I, II and III), each consisting of two subdomains (A and B), and its native SS cross-links. Based on Majorek *et al.*^44^ (Protein Data Bank code 3V03) and Huang *et al.*^60^.

The relative abundance of enzymatically released peptides containing native Cys or (Cys)_2_ residues was calculated for freshly isolated, extensively stored (6 °C, 6 years, dry) and heated (90 °C, 60 min, in excess water) BSA (Table 1). This allowed comparing levels of a given peptide in different digests, but not those of different peptides in the same digest (see below). Storage decreased the level of most peptides with native Cys or (Cys)_2_ residues. Only five of these were present at the same levels before and after storage (Table 1**, peptides 3**–**5, 17**–**18)**, all of which are located in domains IA and IIIA (Fig. 1). BSA contains three domains (I, II and III) each consisting of two subdomains (A and B) with common structural motifs^44^,^45^. It remains to be investigated whether these domains are less easily exposed as a result of storage or heating than other regions. Heating did not significantly change (P > 0.05) the level of the enzymatically released peptide connecting Cys-34 to a free Cys (Table 1, **peptide 3)**. All other peptides with native (Cys)_2_ residues decreased as a result of heating (Table 1**, peptides 1**–**2, 4**–**21)**. The levels of peptides 1 and 2 did not decrease more as a result of heating than as a result of storage. In agreement, extensive dry storage and heating in excess water decreased the level of free SH groups from 44 (±0) mol% to 17 (±2) and 19 (±2) mol%, respectively.

Heating (60 min, 90 °C, excess water) and subsequent enzyme treatment resulted in at least four peptides with non-native (Cys)_2_ residues that were not detected in freshly isolated or stored BSA (Table 3). These peptides involved Cys-90, Cys-91, Cys-101, Cys-244, Cys-245, Cys-475, Cys-476 and Cys-486 which may have been rendered accessible for SS bond reshuffling by heating. That these Cys residues are all present in domains IA, IIA and IIIA supports the hypothesis that the A domains are less easily exposed than the B domains. In the B domains, reactions involving Cys were also observed in freshly isolated and extensively stored samples.

No significant differences (P > 0.05) in the abundance of enzymatically released peptides with non-native Cys or (Cys)_2_ residues were noted between freshly isolated, extensively stored and heated BSA. The reactivity of specific Cys residues can only be investigated based on the decrease in levels of peptides containing native Cys or (Cys)_2_ residues residues, or based on exclusive formation upon heating. Of course, blocking the Cys-34 SH group which most likely initiates all SH-SS interchange and SH oxidation, could be an approach for further studying the importance of this Cys residue.

### Impact of SS bond formation and reshuffling on bovine serum albumin aggregation

**Figure 2:**
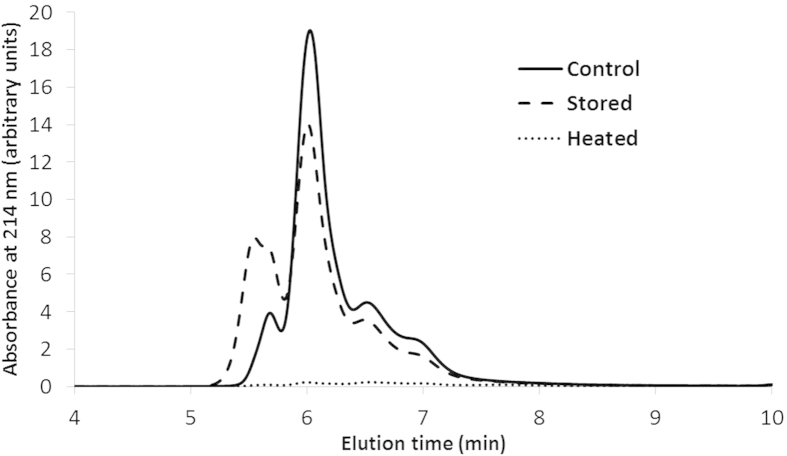
Size exclusion HPLC profiles of bovine serum albumin. Freshly isolated, extensively stored (6 years, 6 °C, dry) and heated (60 min, 90 °C, excess water) samples were extracted in 0.05 mol/L sodium phosphate buffer (pH 6.8) containing 2.0% (w/v) sodium dodecyl sulfate.

The aggregation of BSA was studied by evaluating its molecular weight profile and extractability in sodium dodecyl sulfate (SDS) containing media. Figure 2 shows the size exclusion-HPLC profiles of extracts from unheated, extensively stored, and heated BSA samples in SDS containing medium. During extraction, covalent cross-links between proteins remain intact while non-covalent interactions are impacted by SDS and the sodium phosphate buffer.

The freshly isolated sample mainly contained BSA monomers which eluted at around 6.0 min. The second largest peak in freshly isolated BSA represented BSA oligomers eluting at around 5.6 min. MALDI-MS measurements have demonstrated that BSA forms dimers, trimers, tetramers and pentamers^48^. Based on the elution profiles, we conclude that only BSA and its dimers and trimers were present in the freshly isolated BSA sample, in agreement with Barone *et al.*^49^. The level of free SH groups and presence of various amino acid sequences with non-native Cys or (Cys)_2_ residues in freshly isolated BSA confirmed that SS bond formation and reshuffling during protein isolation induced dimerization and possibly oligomerization. Based on fluorescence resonance energy transfer measurements, Levi and González Flecha^50^ assumed that Cys-34 is not the only Cys residue involved in self-association of BSA. Our results confirm that at least twelve other Cys residues are impacted during protein isolation and are involved in di- and oligomerization.

Storage increased the amount of BSA dimers at the expense of monomers, but it did not change the overall extractability in SDS containing medium. Heating BSA for 60 min at 90 °C in excess water reduced its extractability in SDS containing medium to 4%. Presence of a reducing agent during extraction completely restored the extractability of heated BSA, confirming that SS cross-links largely contribute to the heat-induced extractability loss. SS cross-links result from reshuffling of SS bonds (SH-SS interchange) or formation of new SS bonds (oxidation of free SH groups). Because storage and heat treatment decreased the level of free SH groups to the same extent, equal SH oxidation was assumed. In agreement, both treatments similarly impacted peptides containing Cys-34 with its free SH group (Table 1**, peptides 1 and 2**). Still, storage and heating had a different impact on protein aggregation. Thus, SH-SS interchange reactions probably contributed more to BSA polymerization than SH oxidation, which usually terminates cross-linking^51^.

Given the importance of SS bond reshuffling, we here further investigated the difference in impact of storage and heat treatment on levels of enzymatically released Cys- and (Cys)_2_-containing peptides. Clearly, not the extent of SS bond reshuffling but rather the location of newly formed SS bonds greatly determined protein aggregation. Intramolecular bonds can change protein conformation, while intermolecular bonds change its molecular weight. Eight Cys residues (Cys-90, Cys-91, Cys-101, Cys-244, Cys-245, Cys-475, Cys-476, Cys-486) were involved in SS bond reshuffling during heating but not during isolation or extensive storage. Treatments which cause BSA to precipitate in water expose at least 40 to 50% of the SS bonds, *i.e.* seven to nine (Cys)_2_ residues^41^. It remains to be investigated whether the involvement of these (Cys)_2_ residues in SS bond reshuffling is crucial for protein aggregation.

## Discussion

To the best of our knowledge, this paper is the first to study SS bond formation and reshuffling during protein isolation, storage and heating. Tandem MS with alternating ETD and CID provided information on the reactivity of specific Cys and (Cys)_2_ residues in BSA. Results demonstrated that isolation of BSA from plasma already impacts at least thirteen different Cys residues. Some of the free SH groups (Cys-34) in freshly isolated BSA form SS bonds with free Cys, but no evidence was found for a cross-link between the free SH group of BSA and glutathione. Extensive dry storage, which did not impact the extractability of BSA in SDS containing medium, further decreased the level of most SS bonds present in native BSA, except those in domain IIIA. In contrast, heat treatment drastically reduced the extractability of BSA and decreased the level of all native SS bonds. Results also indicated that some (Cys)_2_ residues in domains IA, IIA and IIIA are only involved in SS bond formation and reshuffling during thermal treatment. These (Cys)_2_ residues may well play a key role during BSA cross-linking.

Evidently, the approach offers a platform for understanding Cys-related PTMs in other proteins, especially since novel data mining and machine learning algorithms for MS-based proteomics constantly increase its feasibility. Increased knowledge on SS bond formation and reshuffling is particularly relevant for enzymes, hormones and other functional proteins in biochemical, medical, pharmaceutical and/or food context. Two points need to be stressed. Firstly, it is important to select the most appropriate enzyme based on current knowledge on enzyme specificity and amino acid sequence. Secondly, targeted MS is recommended. Even though a huge amount of information can be obtained from automated MS runs, complementary targeted MS runs certainly add value.

## Methods

### Materials

BSA (fraction V for biochemistry) was from Sigma-Aldrich (Diegem, Belgium). Its moisture content was 6.5%. Moisture contents were determined in triplicate according to the AACC-I Approved Method 44-19.01^52^. The protein content (N × 6.25) was determined in triplicate as 96.2% on dry matter basis using an adaptation of the AOAC Official Method 990.03^53^, with an automated Dumas protein analysis system (EAS Variomax N/CN, Elt, Gouda, The Netherlands).

### Storage and heat treatment of BSA

Commercial serum albumin isolated from bovine blood plasma was purchased and immediately analyzed as ‘freshly isolated BSA’. This commercial sample was also heated in water (1.0%, w/v) for 60 min at 90 °C and subsequently freeze-dried to obtain ‘heated BSA’ .  The same commercial sample, stored for 6 years at 6 °C in a closed jar, was used as ‘extensively stored BSA’ .

### Sample preparation for MS

BSA samples (1.0 mg protein) were dissolved in 1.0 mL 0.2 mol/L Tris/HCl buffer (pH 6.5) containing 0.2 mmol calcium chloride and 0.1 mg thermolysin. Samples were magnetically stirred at 37 °C, and after 16 h the reaction was stopped by lowering the pH to 2.0 with 3 μL trifluoroacetic acid (100%). During hydrolysis, the pH did not change more than 0.1 unit. The digest was centrifuged at 9600 × *g* for 20 min at 4 °C. The supernatant was collected and purified by solid phase extraction on Strata-X-C devices (200 mg sorbent mass/3.0 mL; Phenomenex, Torrance, CA, USA). Solid phase extraction cartridges were subsequently conditioned with 1.0 mL of methanol and 1.0 mL of trifluoroacetic acid (0.1%, v/v). The digest (1.0 mL) was applied, and the cartridge washed five times with 1.0 mL 50% (v/v) methanol. Peptides were then eluted with 1.0 mL 2.0 mol/L ammonia, freeze-dried and dissolved in 1.0 mL formic acid (0.1%, v/v).

### LC-ESI-MS/MS with alternating CID/ETD

LC-MS experiments were performed on an HCT-Ultra PTM ion trap mass spectrometer (Bruker Daltonics, Bremen, Germany) with alternating CID/ETD detection coupled with an UltiMate 3000 HPLC (Dionex, Idstein, Germany) system equipped with an Aeris PEPTIDE 3.6 m XB-C_18_ column (2.1 × 150 mm; Phenomenex). The MS contained an ion trap with an ESI interface running in the positive mode (capillary voltage: 2400 V; capillary exit voltage: 158.5 V; skimmer voltage: 40 V). Nitrogen was used as drying (8.0 L/min, 325 °C) and nebulizing gas (0.2 MPa). Fluoranthene radical anions for ETD fragmentation were generated in an additional chemical ionization source filled with fluoranthene and methane gas. The mobile phases for LC separation were (A) 0.1% (v/v) formic acid and (B) 0.1% (v/v) formic acid in acetonitrile. The following gradient was used: (i) linear from 0 to 32% B over 80 min; (ii) linear from 32 to 90% B over 5 min; (iii) isocratic at 90% B for 9 min; (iv) linear from 90 to 0% B in 1 min; (v) isocratic at 0% B for 10 min. The flow rate was 0.2 mL/min, injection volume was 5 μL, and column temperature was 30 °C. An auto-MS^2^ mode was used for the alternating CID/ETD fragmentation. After a full-scan MS spectrum from *m/z* 150 to 3000 had been acquired (smart target value 3 × 10^5^ ions or a maximum acquisition time 100 ms), CID-MS^2^ (fragmentation amplitude of 1 V) and ETD-MS^2^ [ion current control (ICC) > 6 × 10^5^ counts] scan steps were performed on the same precursor ion.

### Peptide identification and quantification

To identify BSA peptides with reduced Cys residues, CID fragmentation spectra were screened for parent ions with *m/z* values corresponding to peptides containing one or more free SH groups, both manually and using the Biotools 3.0 software (Bruker Daltonics, Bremen, Germany). To identify SS-linked peptides, fragmentation spectra after ETD which preferentially cleaves SS bonds, were manually screened for fragments with *m/z* values corresponding to Cys-containing peptides. In one study, ETD of highly charged ions from proteins with few cyclic regions resulted in considerable cleavage of peptide instead of SS bonds^54^. However, the ions fragmented in this study were BSA peptides with molecular masses below 3200 and charge states below 5. In addition, 67% of the secondary structure of BSA consists of α-helices^55^. ETD of peptides with low charge states and consisting of one or more small peptide chains with either intra- or interchain SS bonds preferentially cleaves SS bonds^56^. Having identified potential SS-linked peptides based on ETD spectra, their corresponding CID spectra were manually validated according to the principles by Rombouts *et al.*^57^. For ions consisting of x amino acids, at least x-2 isotopically resolved independent fragment peaks were required to match the theoretical peptide fragments. Only a-, b- or y-ions, or associated peaks arising due to water or amine loss were considered as daughter ions of a peptide, and either the b- or y-ion series were required to confirm at least three consecutive amino acids in the peptide sequence. Only one isotopically resolved peak with an intensity higher than one third of the maximum intensity, an *m/z* value larger than that of the double/triple charged parent ion and not matching a theoretical peptide fragment, were allowed. Semi-quantitative determination of peptides was based on Silva *et al.*^58^. Control peptides without Cys from various BSA domains were selected: LSHKDDSPDLPK (Ia-Ib), AIPENLPP (IIa-IIb), LHEKTP (IIIb). Peak areas of detected Cys- and (Cys)_2_-containing peptides in a specific sample were expressed as a percentage of the average of the peak areas of these control peptides in the same sample. This quantitation method allowed comparing different intensity values of a given peptide in different digests (relative concentrations), but not comparing different intensity values of different peptides in the same digest.

### Size exclusion high performance liquid chromatography (HPLC)

The impact of isolating and heating on BSA molecular weight and extractability in SDS containing media was evaluated by size exclusion HPLC using a LC-2010HT system (Shimadzu, Kyoto, Japan) as described by Lagrain *et al.*^59^. Proteins were extracted (1.0 mg protein/mL) from BSA samples with a 0.05 mol/L sodium phosphate buffer (pH 6.8) containing 2.0% (w/v) SDS. Extractability under reducing conditions was determined under N_2_ atmosphere in the same SDS buffer, now also containing 1.0% (w/v) dithiothreitol. All analyses were carried out in duplicate. After centrifugation (10 min, 10000 × *g*) and filtration (Millex-GP, 0.45 μm, polyethersulfone, Millipore, Darmstadt, Germany), samples were loaded (60.0 μL) on a Biosep-SEC-S4000 column with a separation range of 15000 to 500000 (Phenomenex). The gradient was isocratic with an acetonitrile (ACN)/water mixture (1/1, v/v) containing 0.05% (v/v) trifluoroacetic acid at a flow rate of 1.0 mL/min. The column temperature was 30 °C. Detection was at 214 nm. The portion of proteins extractable in SDS containing buffer was calculated from the area under the size exclusion chromatogram and expressed as a percentage of the total extractable proteins, *i.e.* the extractable protein from the freshly isolated BSA under reducing conditions. Relative standard deviations did not exceed 10%.

### Quantification of free SH groups

Free SH groups were determined colorimetrically after reaction with 5,5-dithio-bis(2-nitrobenzoic acid) (DTNB). Samples containing 0.9 to 1.3 mg protein were shaken for 60 min in 1.0 mL sample buffer [0.10 mol/L tris(hydroxymethyl)aminomethane (pH 8.0) containing 2.0% (w/v) SDS, 3.0 mol/L urea and 1.0 mmol/L tetrasodium ethylenediamine tetraacetate]. Then, 100 μL DTNB reagent [0.1% (w/v) in sample buffer] was added and the samples shaken for another 10 min. After filtration over polyethersulfone (0.45 mm, Millex-HP, Millipore) and exactly 45 min after addition of DTNB reagent, the absorbance at 412 nm (path length 10 mm) was read. Absorbance values were converted to levels of free SH using a calibration curve with reduced glutathione^51^. Controls without DTNB or sample were used to correct for background absorbance of DTNB and sample.

### Statistical analyses

Protein extractabilities in SDS containing media and levels of free SH groups were analyzed by one-way analysis of variance using JMP Pro software 11.0.0 (SAS Institute, Cary, NC, USA), with comparison of mean values using the Tukey test (α = 0.05).

## Additional Information

**How to cite this article**: Rombouts, I. *et al.* Formation and reshuffling of disulfide bonds in bovine serum albumin demonstrated using tandem mass spectrometry with collision-induced and electron-transfer dissociation. *Sci. Rep.* **5**, 12210; doi: 10.1038/srep12210 (2015).

## Figures and Tables

**Table 1:**
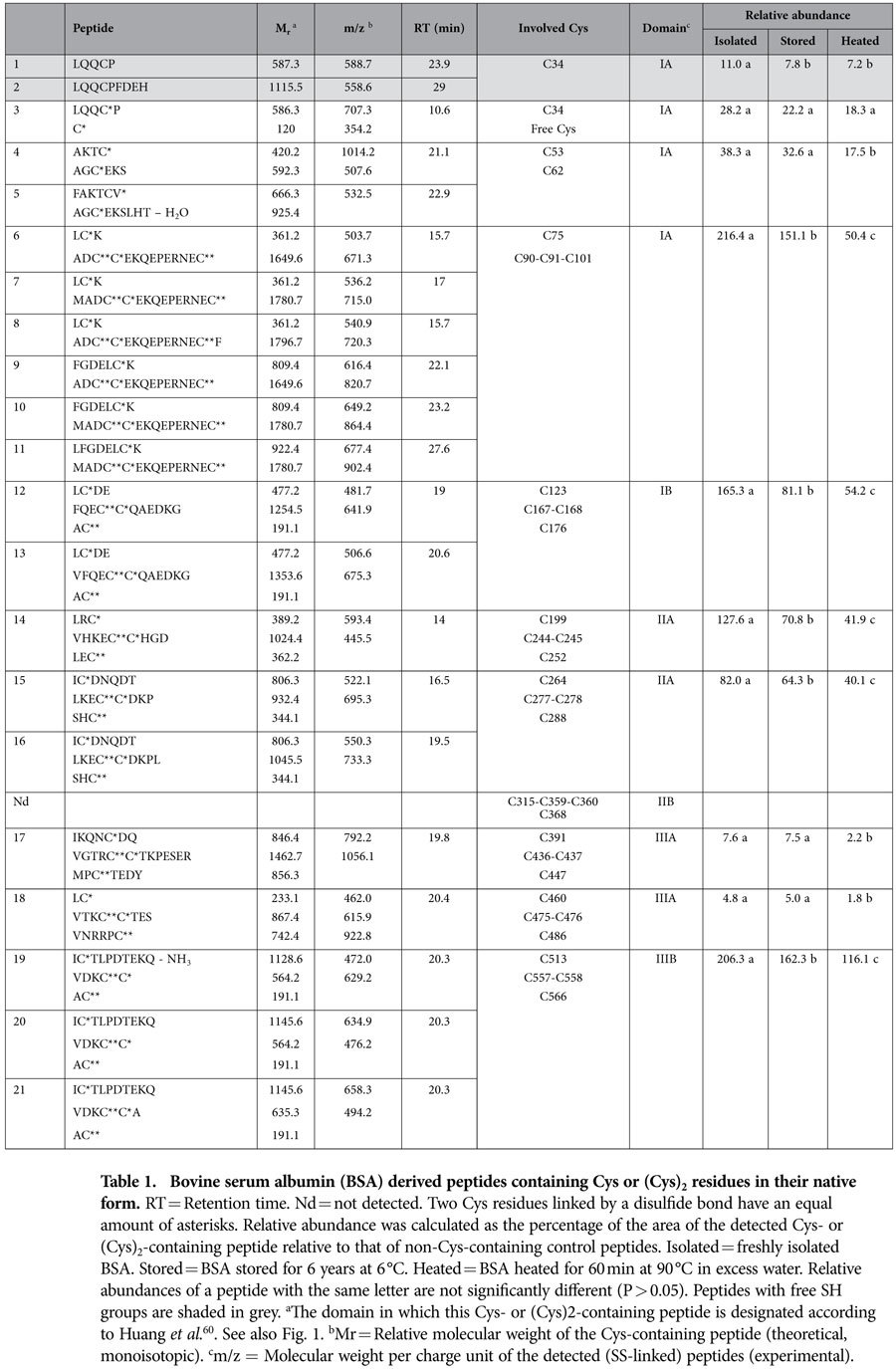
Bovine serum albumin (BSA) derived peptides containing Cys or (Cys)_2_ residues in their native form.

**Table 2:**
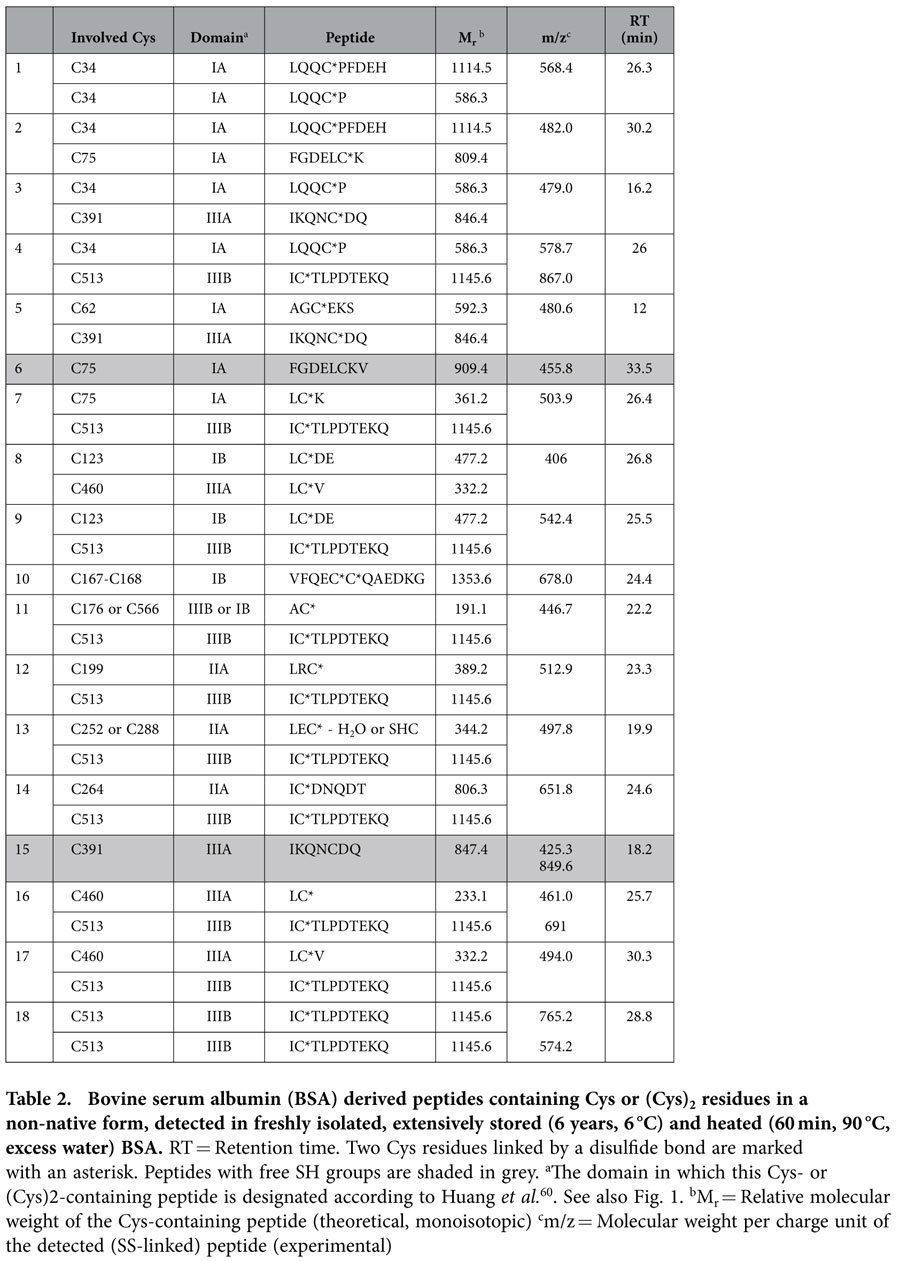
Bovine serum albumin (BSA) derived peptides containing Cys or (Cys)_2_ residues in a non-native form, detected in freshly isolated, extensively stored (6 years, 6 °C) and heated (60 min, 90 °C, excess water) BSA.

**Table 3:** Bovine serum albumin (BSA) derived peptides containing (Cys)_2_ -residues in a non-native form, exclusively detected in heated (60 min, 90 °C, excess water) BSA.

	Involved Cys	Domain^a^	Peptide	M_r_^b^	m/z^c^	RT (min)
1	C90-C91-C101	IA	ADC**C*EKQEPERNEC**F	1796.7	570.2	18.4
	C123	IB	LC*DE	477.2		
2	C123	IB	LC*DE	477.2	407.8	15.6
	C486	IIIA	VNRRPC*	742.4		
3	C244-C245	IIA	VHKEC*C*HGD	1024.4	342	10.1
4	C475-C476	IIIA	VTKC*C*TES	867.4	435	15.1

RT = Retention time. Two Cys residues linked by a disulfide bond are marked with the same amount of asterisks.

^a^The domain in which this (Cys)_2_ -containing peptide is designated according to Huang *et al.*^60^. See also Fig. 1.

^b^M_r_ = Relative molecular weight of the Cys-containing peptide (theoretical, monoisotopic)

^c^m/z = Molecular weight per charge unit of the detected (disulfide-linked) peptide (experimental)
