# Corrigendum: Formation and reshuffling of disulfide bonds in bovine serum albumin demonstrated using tandem mass spectrometry with collision-induced and electron-transfer dissociation

**DOI:** 10.1038/srep15589

**Published:** 2015-10-30

**Authors:** Ine Rombouts, Bert Lagrain, Katharina A. Scherf, Marlies A. Lambrecht, Peter Koehler, Jan A. Delcour

Scientific Reports 5: Article number: 1221010.1038/srep12210; published online: 07202015; updated: 10302015

Marlies A. Lambrecht was omitted from the author list in the original version of this Article.

This has been corrected in the PDF and HTML versions of the Article.

The Author Contributions section now reads:

Conceived and designed the experiments: I.R. and B.L. Conducted the experiments: I.R., B.L., K.A.S., M.A.L. Analyzed the results: I.R. Contributed reagents/materials/analysis tools: P.K. and J.A.D. Wrote the paper: I.R. Reviewed the manuscript: I.R., B.L., K.A.S., P.K. and J.A.D.

